# RGMa and Neogenin control dendritic spine morphogenesis via WAVE Regulatory Complex-mediated actin remodeling

**DOI:** 10.3389/fnmol.2023.1253801

**Published:** 2023-10-19

**Authors:** Kai Sempert, Belal Shohayeb, Vanessa Lanoue, Elizabeth A. O’Brien, Cecilia Flores, Helen M. Cooper

**Affiliations:** ^1^Queensland Brain Institute, The University of Queensland, Brisbane, QLD, Australia; ^2^Department of Psychiatry, McGill University, Montréal, QC, Canada; ^3^Department of Neurology and Neurosurgery, McGill University, Montréal, QC, Canada; ^4^Douglas Mental Health University Institute, Montréal, QC, Canada

**Keywords:** spine enlargement, actin cytoskeleton, synapse formation, Neogenin, RGMa, netrin receptor, WAVE Regulatory Complex

## Abstract

Structural plasticity, the ability of dendritic spines to change their volume in response to synaptic stimulation, is an essential determinant of synaptic strength and long-term potentiation (LTP), the proposed cellular substrate for learning and memory. Branched actin polymerization is a major force driving spine enlargement and sustains structural plasticity. The WAVE Regulatory Complex (WRC), a pivotal branched actin regulator, controls spine morphology and therefore structural plasticity. However, the molecular mechanisms that govern WRC activation during spine enlargement are largely unknown. Here we identify a critical role for Neogenin and its ligand RGMa (Repulsive Guidance Molecule a) in promoting spine enlargement through the activation of WRC-mediated branched actin remodeling. We demonstrate that Neogenin regulates WRC activity by binding to the highly conserved Cyfip/Abi binding pocket within the WRC. We find that after Neogenin or RGMa depletion, the proportions of filopodia and immature thin spines are dramatically increased, and the number of mature mushroom spines concomitantly decreased. Wildtype Neogenin, but not Neogenin bearing mutations in the Cyfip/Abi binding motif, is able to rescue the spine enlargement defect. Furthermore, Neogenin depletion inhibits actin polymerization in the spine head, an effect that is not restored by the mutant. We conclude that RGMa and Neogenin are critical modulators of WRC-mediated branched actin polymerization promoting spine enlargement. This study also provides mechanistic insight into Neogenin’s emerging role in LTP induction.

## 1. Introduction

The ability of the excitatory synapse to undergo long-lasting changes in structure and composition is correlated with increased synaptic strength and is essential for synaptic plasticity. Dendritic spines are highly dynamic and undergo rapid morphological changes in response to synaptic activity (structural plasticity). Long-term potentiation (LTP), the cellular correlate of learning and memory, induces both spinogenesis and spine enlargement (Matsuzaki et al., 2004; Harvey and Svoboda, 2007; Bourne and Harris, 2011; Watson et al., 2016). LTP triggers branched actin polymerization adjacent to the post-synaptic density, thereby promoting spine growth and the consolidation of the potentiated state (Matsuzaki et al., 2004; Okamoto et al., 2004; Honkura et al., 2008; Bosch et al., 2014). Actin remodeling is therefore a key driver of spine formation and structural plasticity.

Spine formation and enlargement in response to synaptic activity is dependent on the Arp2/3 complex, an inefficient actin nucleator which catalyzes branched actin formation (Hotulainen et al., 2009; Korobova and Svitkina, 2010; Spence et al., 2016). Arp2/3 is activated by the pentameric WAVE Regulatory Complex (WRC: Cyfip1, Nckap1, WAVE, Abi1/2, HSPC300), a pivotal branched actin regulator controlling spine morphogenesis (Kim et al., 2006; Chen et al., 2014; Spence et al., 2016). Mutation or loss of WRC subunits results in spine depletion and impairment of synaptic transmission and plasticity, leading to memory and learning deficits in mice (Grove et al., 2004; Kim et al., 2006; Soderling et al., 2007; De Rubeis et al., 2013; Hazai et al., 2013; Pathania et al., 2014; Davenport et al., 2019). Although tight regulation of the WRC is critical for spine morphogenesis and synaptic activity, we currently have little understanding of the molecular mechanisms that spatiotemporally regulate WRC activity - a requirement for Arp2/3-mediated branched actin nucleation.

Neogenin was initially described as an axon guidance receptor for the Repulsive Guidance Molecules (RGMa,b,c) and an attractive receptor for Netrin-1 (Keeling et al., 1997; Rajagopalan et al., 2004; Wilson and Key, 2006; De Vries and Cooper, 2008). It is also recognized as a crucial regulator of nervous system development, where it plays a central role in cortical progenitor function, neurogenesis, neuronal migration and gliogenesis (Tassew et al., 2012; O'Leary et al., 2013, 2015; van Erp et al., 2015; Huang et al., 2016; Huang and Xiong, 2016; Kam et al., 2016). Moreover, it is now emerging as a critical component of the excitatory post-synaptic apparatus regulating synaptic plasticity. Genetic deletion of Neogenin in immature granule cells of the dentate gyrus impairs synaptic transmission, dendrite growth and branching, leading to depressive-like behavior (Sun D. et al., 2018). In addition, after conditional deletion of Neogenin in embryonic neural progenitors, mature pyramidal neurons of the basolateral amygdala fail to generate mature spines, do not exhibit LTP induction and maintenance, and have a deficit in fear memory retrieval (Sun X.-D. et al., 2018). It should however be noted that a defect in astrocyte function may, at least in part, contribute to this phenotype in these mice. Neogenin is also essential for the induction of LTP at the entorhinal to granule cell synapse of the perforant path (Liakath-Ali et al., 2022). Together these studies argue that the Neogenin signaling pathway directly impacts synaptic plasticity in excitatory neurons. However, the post-synaptic mechanism through which Neogenin acts to promote plasticity within the spine has yet to be explored.

Our previous studies revealed that Neogenin anchors the WRC and Arp2/3 to restricted sites of cadherin adhesion due to its ability to directly bind a highly conserved pocket within the WRC comprising the Cyfip1 and Abi subunits, thereby spatiotemporally controlling branched actin polymerization (Lee et al., 2016; O'Leary et al., 2017). Here we test the hypothesis that Neogenin and its ligand RGMa are critical modulators of WRC-mediated branched actin polymerization driving spine enlargement in the hippocampus.

## 2. Materials and methods

### 2.1. Primary hippocampal neuronal culture

Primary hippocampal neuronal cultures were prepared from C57BL/6 J embryos at embryonic day 18.5 (E18.5) as previously described (Lanoue et al., 2017). Briefly, hippocampi were dissected and dissociated via proteolytic digestion in trypsin solution at 37°C for 20 min followed by gentle trituration. Dissociated cells in Neurobasal medium (Gibco) containing 2% B27-Supplement (Gibco) and 0.5 mM L-glutamine (Gibco) were plated on poly-L-lysine (Sigma) and laminin (Gibco) coated 12 mm glass coverslips (4.5 × 10^4^ cells/well, 24 well plate) or directly onto coated wells of 6 well plates (18 × 10^4^ cells/well). Cultures were maintained at 37°C and 5% CO_2_ with 50% medium replacement every 3 days. All experiments involving animals were approved by the Anatomical Biosciences Animal Ethics Committee of the University of Queensland and performed in accordance with the Australian Code of Practice for the Care and Use of Animals for Scientific Purposes.

### 2.2. Constructs and shRNA

Full-length mouse Neogenin or zebrafish Neogenin (Neo) (6 x myc epitopes added to the C-terminus) or mouse RGMa (N-terminal myc-tag) were cloned into pCS2+ and then subcloned into pCAGIG which includes an IRES-GFP (green fluorescent protein) sequence to visualize neurons. As mouse and zebrafish Neogenin differ in their nucleotide sequences at the shRNA target sites, the zebrafish cDNA was used in rescue experiments as previously described (Lee et al., 2016). RNAi resistant mouse RGMa constructs were generated by mutating 4 nucleotides within the shRNA target sequence using the QuickChange II XL Site-Directed Mutagenesis Kit (Agilent Technologies) according to the manufacturer’s instructions. For the mutant NeoΔWIRS constructs, amino acids S1314 and F1315 (NCBI: AY082380.1) were mutated to alanine using the QuickChange II XL Site-Directed Mutagenesis Kit (Lee et al., 2016). The extracellular and transmembrane domains of zebrafish Neo and NeoΔWIRS were replaced with a myristoylation sequence (MGSSKSKPKDPS) placed upstream of amino acid 1,088 to generate Neo-ICD and Neo-ICDΔWIRS (O'Leary et al., 2017). shRNAs were expressed using the BLOCK-iT RNA-Polymerase II miRNA expression vector system (ThermoFisher Scientific) which co-cistronically expresses Emerald GFP under the control of the CAG-promoter. In these constructs the shRNA sequence is embedded in the 3’ UTR of GFP, ensuring that the shRNAs are co-expressed with GFP. shRNA sequences: shNeo12:TAATCTTGCCGTTAGCTTCAG, shNeo15:TTATAGTCCACTTTGATGGTCA, shRGMa919:TAATTATTGTCGATGAGAGGC, shRGMa1573:AACAGATGCAGCTTGTCCTTG, shControl:TGCGCGTGGAGAC.

### 2.3. RNAi experiments

At 3 days *in vitro* (DIV3) for dendrite analysis or at DIV12 for spine analysis (Pathania et al., 2014; Spence et al., 2016), hippocampal neurons were transfected with plasmids encoding shRNAs, Neogenin or RGMa using the Lipofectamine 2000 Transfection Reagent (Invitrogen) according to the manufacturer’s instructions. For 24-well or 6-well plates, a total of 0.75 μg or 3 μg DNA, respectively, was used for transfection. After 1.5 h the medium was replaced by an equal volume of fresh and conditioned medium and cultured for an additional 48 h. At 24 h post transfection, recombinant RGMa (recRGMa, Enzo Life Sciences) was added for 24 h at a final concentration of 20 μg/ml (Tassew et al., 2012).

### 2.4. Image acquisition and analysis

For dendritic arbor tracing, GFP-positive neurons were randomly selected and single-plane images at 20x were acquired using an epifluorescence microscope (Axio imager, Zeiss). The entire dendritic arbor of each neuron was traced based on the GFP signal using the NeuronJ plugin from FIJI (National Institutes of Health, USA). Neurites shorter than 10 μm were excluded from the analysis. Primary dendrites were defined as those originating from the soma whereas secondary and tertiary branches extended from the primary and secondary dendrite, respectively. Data were collected from 60 neurons/experimental group over 3 independent experiments.

### 2.5. Spine analysis

For spine analysis, GFP-positive neurons were randomly selected and z-stacks (z-step size 110 nm) obtained using a Nikon Plan Apochromat 100x/1.45 NA oil-immersion objective on a spinning disk confocal microscope (Diskovery, Andor Technology/Nikon, Ti-E microscope body). The acquired images were processed by deconvolution using Huygens Professional (Scientific Volume Imaging). Tracing was performed using Neurolucida 360 (MBF Bioscience) on primary, secondary and tertiary dendrites where 3–5 dendrites/neuron and ~15 neurons/condition were traced using the smart manual tracing mode over 3 independent experiments. The total length of traced dendrites was 300–400 μm with the initial and terminal segments of the dendritic arbor excluded from the analysis. Spines were automatically detected using the following Neurolucida parameter set: outer range, 7.5 μm; minimum height, 0.3 μm; detector sensitivity, 150; minimum count, 35. Spines were classified using the following parameter set: head-to-neck ratio, 1:1.2; length-to-head ratio 1:2.5; head size 0.6 μm; filopodium length 3.5 μm. Obvious software mis-assignments were manually corrected. Experiments were performed blind wherever possible. In pilot experiments we quantified the number of mushroom and thin spines 2 and 4 days after Neogenin shRNA transfection and found no difference in spine numbers. We therefore chose to conduct our analysis at day 2 after transfection (data not shown).

### 2.6. Analysis of shRNA efficiency

Lentivirus was produced in HEK293T cells by co-transfection of FUGW plasmids (gift from David Baltimore, Addgene plasmid #14883, Lois et al., 2002), containing shRNA or cDNA sequences with the pMD2.G, pRSV-Rev and pMDLg/pRRE plasmids (gifts from Didier Trono, Addgene plasmids #12259, 12553, 12251, Dull et al., 1998) using the Lipofectamine 3,000 Transfection Reagent (Invitrogen) according to the manufacturer’s protocol. The transfection medium (OptiMEM, Gibco) was changed after 5.5 h to DMEM (Gibco) containing 10% FCS and 10 mM sodium butyrate. Lentivirus was harvested 48 h later and stored at −80°C. Primary hippocampal neurons were transduced at DIV8 and cultured until DIV14 (70–75% efficiency). Neurons were then lysed in KALB lysis buffer (150 mM sodium chloride, 50 mM Tris pH 7.4, 1 mM EDTA pH 8, 1% Triton X-100, 10% glycerol, EDTA-free Complete Inhibitors, Roche) at 4°C for 45 min. Samples were separated by SDS-PAGE using NuPAGE 4–12% Bis-Tris Protein Gels (Invitrogen) and transferred onto Immobilon®-FL PVDF-membranes (Merck-Millipore). After blocking for 1 h (Intercept Blocking Buffer, Li-COR), membranes were incubated with primary antibodies (Intercept Blocking Buffer; 0.1% Tween 20) at room temperature (RT) for 1 h and then with secondary antibodies (RT, 1 h). Primary antibodies: Neogenin, AF1079, R&D Systems, 1:200; GAPDH, CSB-PA00025A0rb, Cusa Biotechnology, 1:1000; ab3280, Abcam, 1:1000. Secondary antibodies: IRDye® 680LT donkey anti-goat IgG, LI-COR, 1:20000; IRDye® 800CW donkey anti-mouse IgG, LI-COR, 1:15000. Proteins were visualized using an Odyssey scanner system including LI-COR scanning software. Densiometric analysis was performed using FIJI and Adobe Photoshop (Adobe Inc.). Data represent the mean of 3 independent experiments.

HEK293T cells were cultured in DMEM +10% FCS and transfected at 50% confluency using Lipofectamine 2000. For 6-well plates, a total of 1 μg DNA and 7.5 μl cold Lipofectamine in 500 μl warm OptiMEM medium was used for transfection. The transfected cells (≥95% efficiency) were incubated at 37°C and 5% CO_2_ for 24 or 48 h before preparation of lysates and western blotting as described above. Primary antibodies: Neogenin, AF1079, R&D Systems,1:200; β-actin, ab3280, 1:1000; RGMa, sc-67052, Santa Cruz Biotechnology, 1:200. Secondary antibodies as above.

### 2.7. Immunocytochemistry

Hippocampal neurons on 12 mm glass coverslips were fixed in 2% paraformaldehyde (RT, 20 min), blocked in 4% donkey serum in PBS + 0.2% Triton X-100 (RT, 1 h) and incubated with primary antibodies (Neogenin, AF1079, R&D Systems,1:200; RGMa, sc-67052, Santa Cruz Biotechnology, 1:200; Cyfip1, Upstate #07–531, 1:400; WAVE1, Biolegend, #817901; GFP, ab13970, Abcam, 1:1000; myc, 9E10, Sigma-Aldrich, 1:1000). After the PBS wash steps, cells were incubated with fluorophore-conjugated secondary antibodies (Alexa anti-chicken 488, 1:1000; Alexa anti-mouse 546, 1:1000; Alexa anti-goat 568, 1:1000; Alexa anti-rabbit 647, 1:1000; Invitrogen) and mounted onto glass coverslips using ProLong Gold Antifade Mountant (Invitrogen). Images were acquired on an LSM 710 Zeiss confocal microscope and analysis was performed in ImageJ.

### 2.8. F-actin polymerization analysis

F:G actin ratio: F-actin was labeled with phalloidin-Alexa 647 (Invitrogen, A22278) and G-actin was labeled with DNase I-Alexa 594 (ThermoFisher) (O'Leary et al., 2017). Neurons were imaged on a Zeiss LSM 710 confocal microscope. The mean fluorescence intensities of G- and F-actin were quantified in spine heads using ImageJ.

Fluorescence resonance energy transfer (FRET) analysis: hippocampal neurons were co-transfected with cDNAs encoding YFP-actin and CFP-actin (Okamoto et al., 2004) at a ratio of 3:1 with shControl or shNeo12 (with co-cistronic GFP) at DIV12. At DIV14 live neurons were imaged in a phenol red-free medium (125 mM NaCl, 2.5 mM KCl, 25 mM HEPES pH 7.4, 33 mM glucose, 1 mM MgCl_2_). Initially, the spectral overlap between CFP, GFP and YFP emission profiles was determined by expressing each fluorescent protein alone and acquiring the emission spectrum for each fluorophore after excitation using both 458 nm (50%) and 514 nm (2%) laser lines of an Argon laser (Lasos) with a spectral detector on a Zeiss LSM710 confocal microscope (Lambda Mode, Zen Black 2012, Carl Zeiss Pty Ltd., Australia). Using the individual spectral profiles, the overlap between spectra was separated by performing linear spectral unmixing with Zen Black software. After co-transfection of CFP-actin, YFP-actin and shRNAs into neurons, FRET was quantified by acquiring the entire emission spectra of the donor (CFP; excitation, 458 nm; emission, 473 nm) and acceptor (YFP; excitation, 514 nm; emission, 529 nm) fluorophores followed by spectral separation (466–587 nm). The mean fluorescence intensities for CFP-actin and YFP-actin were quantified in spine heads using ImageJ and the YFP:CFP fluorescence intensity ratio calculated after subtraction of the baseline fluorescence for each fluorophore. The Ratio Plus ImageJ plugin was used to generate the YFP:CFP ratio images. The YFP-actin and CFP-actin constructs were a gift from Kenichi Okamoto (Lunenfeld-Tanenbaum Research Institute) and Yasunori Hayashi (Kyoto University).

### 2.9. Experimental design and statistical analysis

Statistical analyses were performed using GraphPad Prism (GraphPad software; 8.3.1). All data were tested for normality (Gaussian distribution) using the Kolmogorov–Smirnov test. Statistical significance was then tested using an unpaired Student *t*-test for two group comparisons. One-way ANOVA followed by the Tukey’s multiple comparisons *post hoc* test was used to compare three or more groups when data were normally distributed. The Kruskal–Wallis test followed by Dunn’s *post hoc* test was used for non-parametric data. All values are presented as mean ± standard error of the mean (SEM). Statistical significance was considered to be *p* < 0.05.

## 3. Results

### 3.1. Neogenin is essential for dendritic spine morphogenesis

**Figure 1:**
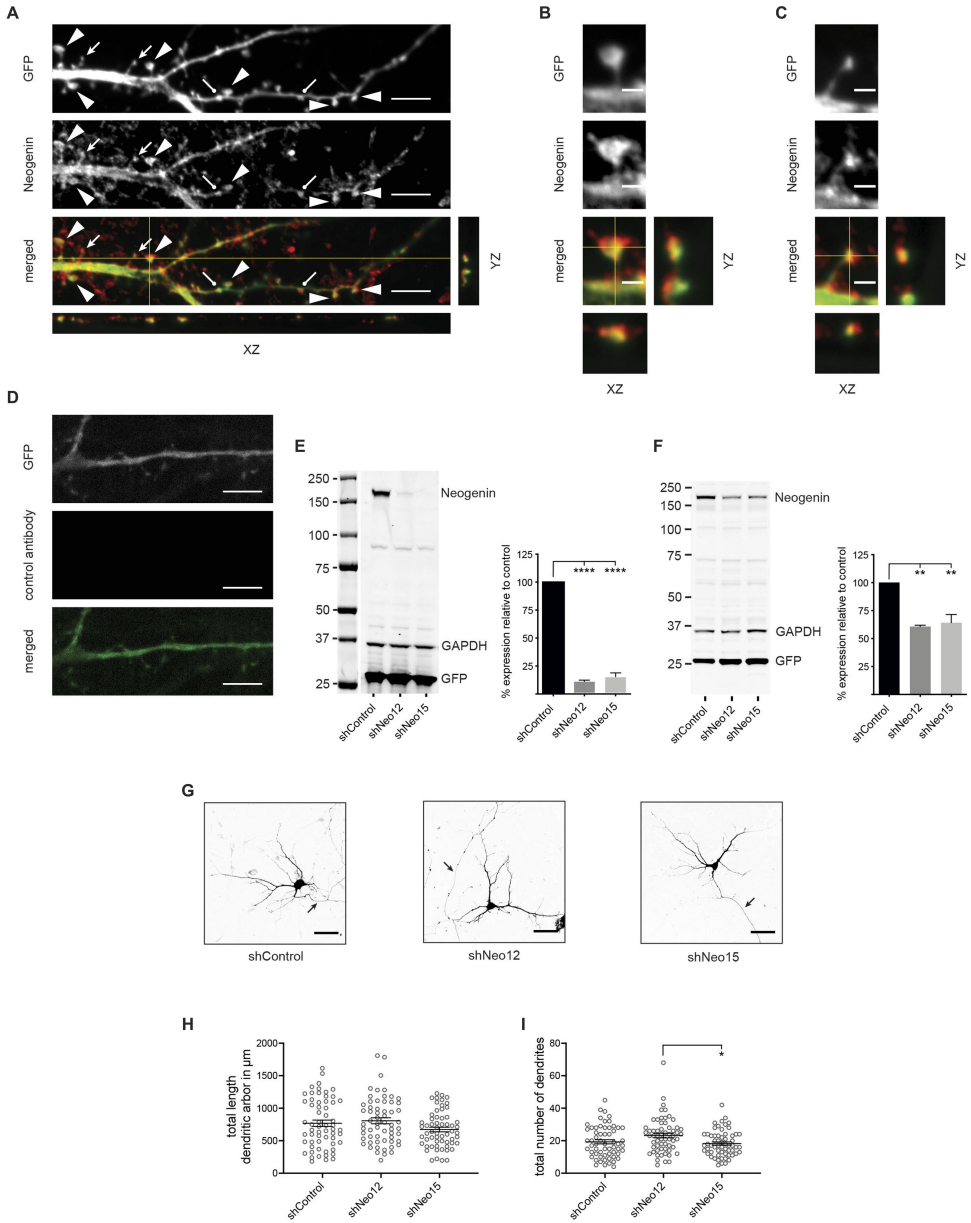
Neogenin is expressed in hippocampal neurons, but its depletion does not affect dendritic growth. **(A)** Endogenous Neogenin was localized to dendritic spines (arrowheads, mushroom spines; filled arrows, thin spines; round-headed arrows, stubby spines) and was detected in the heads of mushroom spines **(B)** and the tips of thin spines **(C)**. **(D)** Isotype-matched IgG antibodies confirmed Neogenin antibody specificity. **(E,F)** Immunoblots: shRNAs efficiently reduced Neogenin expression in HEK293T cells [*n* = 4, one-way ANOVA, Tukey’s *post hoc* test; shNeo12, shNeo15, *p* < 0.0001; *F*_(2,9)_ = 300.5, *p* < 0.0001] **(E)** and hippocampal neurons [*n* = 3, one-way ANOVA, Tukey’s *post hoc* test, shNeo12, *p* < 0.0018; shNeo15, *p* < 0.0013; *F*_(2,6)_ = 26.33, *p* = 0.0011] **(F)**. **(G)** Representative images of neurons transfected with GFP and shNeo12 or 15. Depletion of Neogenin did not affect dendritic outgrowth [*F*_(2,177)_ = 2.47, *p* = 0.0875] **(H)** or branching [*F*_(2,177)_ = 4.463, *p* = 0.0129] **(I)**, *n* = 60 neurons, 3 independent experiments, one-way ANOVA, Tukey’s *post hoc* test. **(E,F,H,I)** Mean ± SEM, *p < 0.05, **p < 0.01, ****p < 0.0001. Scale bars: **(A,D)** 5 μm; **(B,C)** 1 μm; **(G)** 50 μm.

Neogenin is expressed throughout the hippocampus, including the dentate gyrus (Gad et al., 1997; Liakath-Ali et al., 2022). To investigate the hypothesis that Neogenin is a key regulator of dendritic spine morphogenesis we first determined its localization in hippocampal neurons isolated from E18.5 C57BL/6 embryos after transfection with GFP at 12 days *in vitro* (DIV12). Immunolabeling with antibodies against Neogenin followed by confocal microscopy at DIV14 revealed that Neogenin was present along dendritic shafts and on all spine types (Figure 1A). High magnification images clearly demonstrated that Neogenin was enriched in the heads of mature mushroom spines and the distal tips of precursor thin spines (Figures 1B,C). Lack of staining with irrelevant, isotype-matched control antibodies demonstrated specificity (Figure 1D).

To investigate the role of Neogenin in dendritogenesis and spine enlargement we employed an RNA interference (RNAi) approach using Neogenin shRNAs (shNeo12 or shNeo15) or an unrelated control shRNA (shControl) containing co-cistronic GFP. To demonstrate specificity HEK293T cells were co-transfected with the shRNAs and wildtype mouse Neogenin cDNA. Western blotting showed that Neogenin expression was decreased by 88% (shNeo12) or 85% (shNeo15) (Figure 1E). In addition, lentiviral transduction of Neo12 and Neo15 shRNAs into cultured hippocampal neurons reduced the expression of endogenous Neogenin by 40% (shNeo12) and 36% (shNeo15) (Figure 1F).

To test if Neogenin is required for dendritic outgrowth and arborisation we depleted Neogenin by transfecting plasmids containing shRNAs and GFP into hippocampal neurons at DIV3 and analyzed dendritic growth by tracing GFP-positive dendrites at DIV5 (Lanoue et al., 2017). Dendritic number, length and branching were unaffected by shRNA depletion of Neogenin (Figures 1G–I). This is in contrast to a previous study showing that dendrite length is reduced in Neogenin null neurons (Sun D. et al., 2018), suggesting that the level of Neogenin remaining in the shRNA-depleted neurons was sufficient to induce dendrite growth.

**Figure 2:**
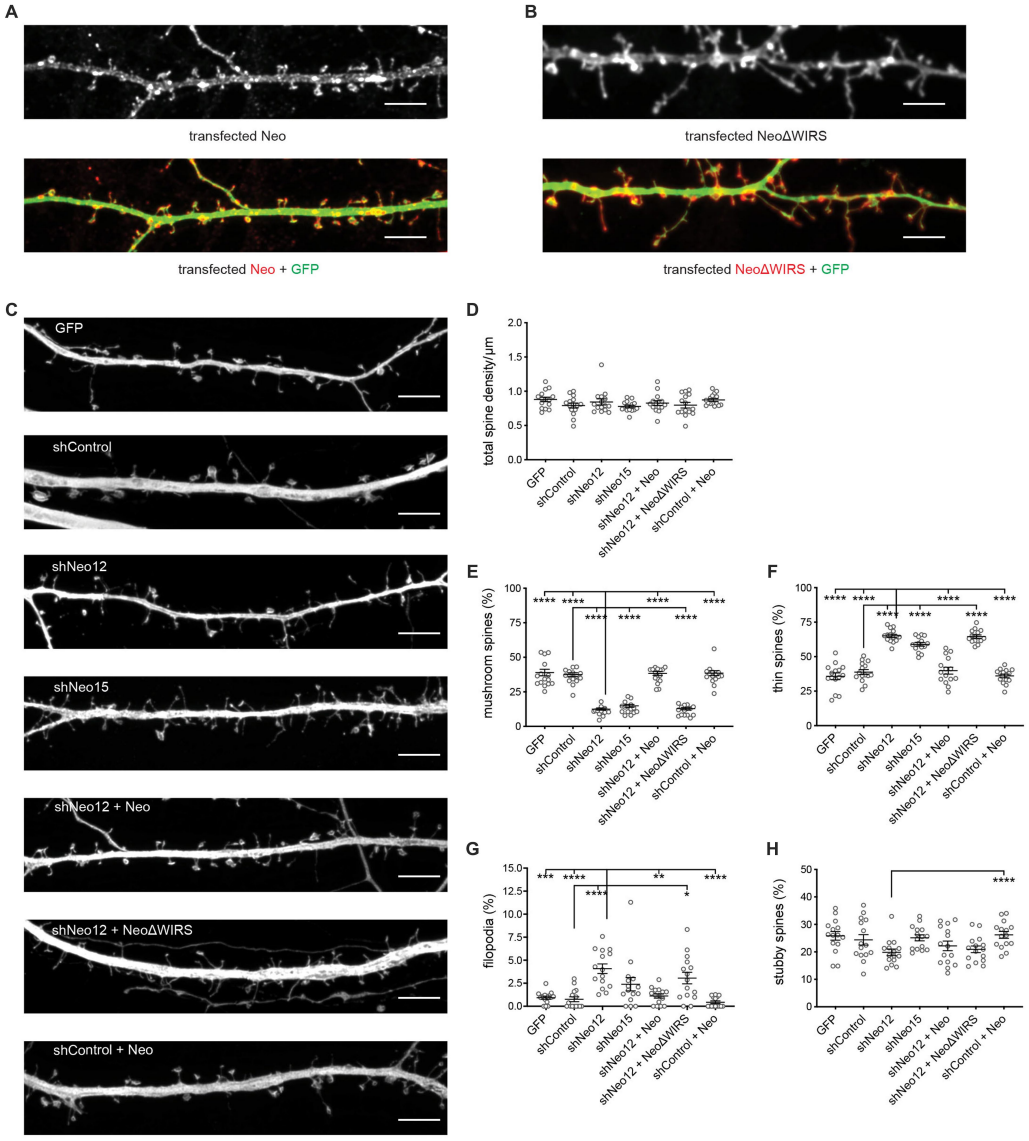
Loss of Neogenin impairs dendritic spine enlargement. shRNA-resistant myc-tagged Neogenin (Neo, red) **(A)** and NeoΔWIRS (red) **(B)** were concentrated in the heads of mushroom and stubby spines as well as in thin spines and filopodia (GFP, green). **(C)** Representative images of hippocampal neurons transfected with GFP, shNeo and Neo or NeoΔWIRS. Depletion of Neogenin did not affect spine density [*F*_(6,98)_ 1.408, *p* = 0.2191] **(D)**, but decreased the proportion of mushroom spines [*F*_(6,98)_ = 81.40, *p* < 0.0001] **(E)** and increased the proportion of thin spines [*F*_(6,98)_ = 60.19, *p* < 0.0001] **(F)** and filopodia (*p* < 0.0001) **(G)**. This phenotype was rescued by Neo but not NeoΔWIRS. The proportion of stubby spines **(H)** was not affected [*F*_(6,98)_ = 2.977, *p* = 0.0103]. *n* = 15 neurons, 3 independent experiments. Mushroom, thin, stubby spines: one-way ANOVA, Tukey’s *post hoc* test; Filopodia, Kruskal–Wallis test, Dunn’s *post hoc* test. **(D–H)** Mean ± SEM. *p < 0.05, **p < 0.01, ***p < 0.001, ****p < 0.0001. Scale bars: **(A–C)** 5 μm.

During spine morphogenesis, filopodia the early precursors of dendritic spines, convert to thin spines upon contact with the pre-synaptic axon, triggering spine head enlargement and the transition to mature mushroom spines (Ziv and Smith, 1996; Lohmann and Bonhoeffer, 2008; Yuste, 2013). Spine enlargement and functional maturation are dependent on WRC-mediated branched actin nucleation (Kim et al., 2006; Soderling et al., 2007; De Rubeis et al., 2013; Chazeau et al., 2014). As Neogenin is known to control the WRC-Arp2/3 pathway (Lee et al., 2016; O'Leary et al., 2017), we tested whether Neogenin was required for spine growth. Neo12 or Neo15 shRNA, along with RNAi-resistant Neogenin (Neo), were transfected into hippocampal neurons at DIV12 and spine density and morphology analysed 2 days later. As seen for endogenous Neogenin, overexpressed Neo was concentrated in the heads of mushroom and stubby spines as well as in filopodia and the distal tips of thin spines (Figure 2A). Assessment of spine density (mushroom, stubby and thin spines, filopodia) along the dendrite demonstrated that Neogenin depletion did not reduce the total number of spines (Figures 2C,D). Depletion of Neogenin, however, resulted in a highly significant 63 to 69% reduction in mature mushroom spines and a concomitant 52–68% increase in the proportion of immature thin spines (shNeo12, shNeo15, respectively; Figures 2C,E,F). We also observed a 5-fold increase in filopodia (shNeo12, Figures 2C,G). In contrast, the proportion of stubby spines was not changed (Figures 2C,H). RNAi specificity was confirmed by co-transfection of shNeo12 with Neo cDNA which restored the number of filopodia, thin and mature spines to normal levels whereas the expression of Neo in shControl neurons had no effect. Rescue with Neo cDNA resulted in a 3.3-fold increase in mature spines, a 1.6-fold reduction in thin spines and a 3.7-fold decrease in filopodia relative to Neo12 shRNA alone (Figures 2C,E–G). Neogenin therefore promotes the progression from filopodia to mature mushroom spines.

### 3.2. Neogenin-WRC interactions promote spine enlargement via branched actin polymerization

**Figure 3:**
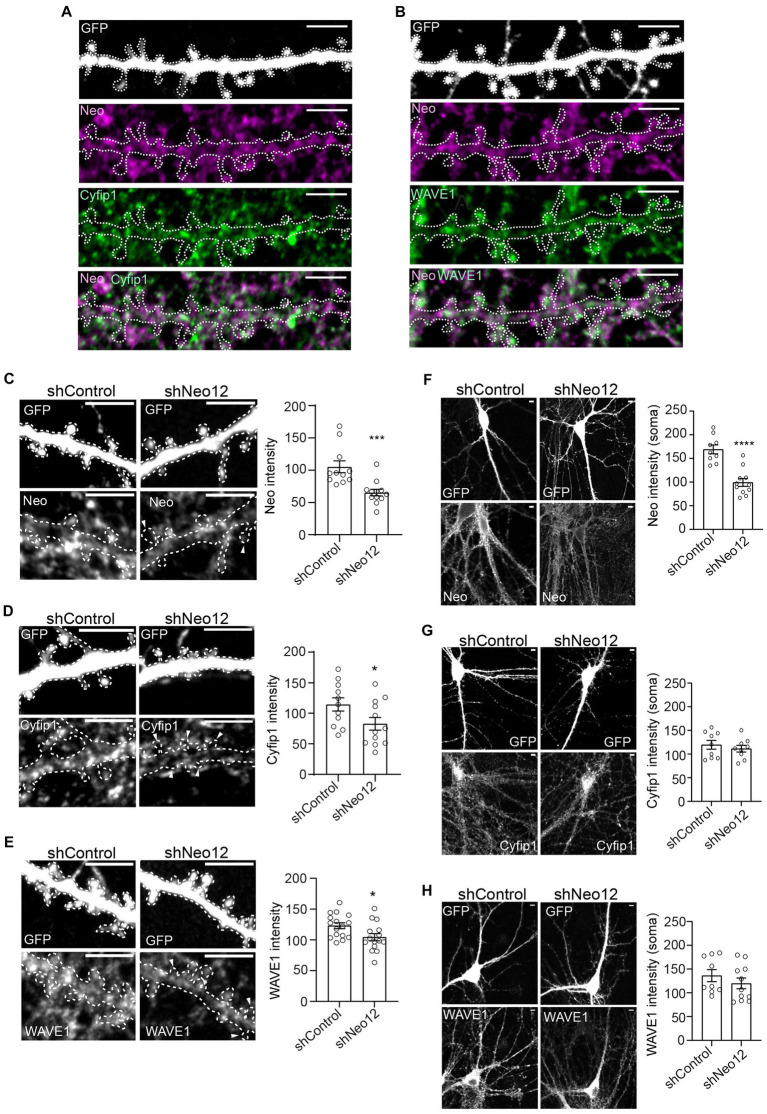
Neogenin depletion reduces Cyfip1 and WAVE1 in hippocampal spines. Co-labeling with antibodies against Neogenin and the WRC subunits Cyfip1 **(A)** or WAVE1 **(B)** demonstrated that endogenous Neogenin colocalizes with the WRC in all spines along the dendrite (*n* = 3). Representative images and quantification of endogenous Neogenin **(C)**, Cyfip1 **(D)** and WAVE1 **(E)** protein levels in spines after expression of shNeo12. Expression of shNeo12 reduces endogenous Neogenin, Cyfip1 and WAVE1 in spines by 38% [*F*_(10,11)_ = 1.024, *p* = 0.0009], 28% [*F*_(10,11)_ = 2.814, *p* = 0.0486] and 16% [*F*_(14, 15)_ = 1.444, *p* = 0.0198], respectively. Representative images and quantification of endogenous Neogenin **(F)**, Cyfip1 **(G)**, and WAVE1 **(H)** protein levels in neuronal cell bodies after expression of shNeo12. Expression of shNeo12 reduces endogenous Neogenin by 41% [*F*_(8,10)_ = 1.064, *p* < 0.0001], but not Cyfip1 [*F*_(8,8)_ = 1.673, *p* = 0.4915] or WAVE1 [*F*_(8,10)_ = 1.001, *p* = 0.3486] in the cell body. *n* = 11–12 neurons, 3 independent experiments, Student’s *t*-test, mean ± SEM. *p < 0.05, ***p < 0.001, ****p < 0.0001. Scale bars: **(A–H)** 5 μm.

The maturation and expansion of mushroom spines is reliant on WRC-Arp2/3-mediated branched actin polymerization (Kim et al., 2006; Soderling et al., 2007; Chazeau et al., 2014). The WRC interacting receptor sequence (WIRS) found in the Neogenin cytoplasmic domain binds to a highly conserved pocket within the WRC which forms only when the full pentameric complex is assembled (Chen et al., 2014; Lee et al., 2016). Within the holocomplex the WIRS domain directly interacts with the Cyfip and Abi subunits (Chen et al., 2014). In line with this, endogenous Neogenin was found to tightly co-localize with both Cyfip1 and WAVE1 in all spines present on hippocampal dendrites (Figures 3A,B). In addition, quantification of the fluorescence intensities of endogenous Neogenin, Cyfip1 and WAVE1 protein levels in spines after expression of shNeo12 indicated that Neogenin depletion (38% decrease) was correlated with loss of Cyfip1 (28% decrease) and WAVE1 (16% decrease) (Figures 3C–E). Although shNeo12 also reduced Neogenin protein levels in the cell body by 41%, Cyfip1 and WAVE1 were unaffected (Figures 3F–H), suggesting that Neogenin is responsible for the recruitment of the WRC to the spine membrane.

To demonstrate that Neogenin-WRC interactions are required for spine enlargement we also attempted to rescue the Neo RNAi-induced maturation phenotype by co-transfecting NeoΔWIRS (RNAi-resistant) which carries mutations in the WIRS motif (Lee et al., 2016; O'Leary et al., 2017). Overexpressed NeoΔWIRS and wildtype Neo were found to localize to thin and mushroom spines at equivalent levels (Figures 2A,B). However, in contrast to wildtype Neo, NeoΔWIRS was unable to rescue the spine maturation defect induced by Neo shRNA as indicated by the significant reduction in mushroom spines (68%) and concomitant increase in thin spines (66%) and filopodia (4-fold) (Figures 2C,E–G).

**Figure 4:**
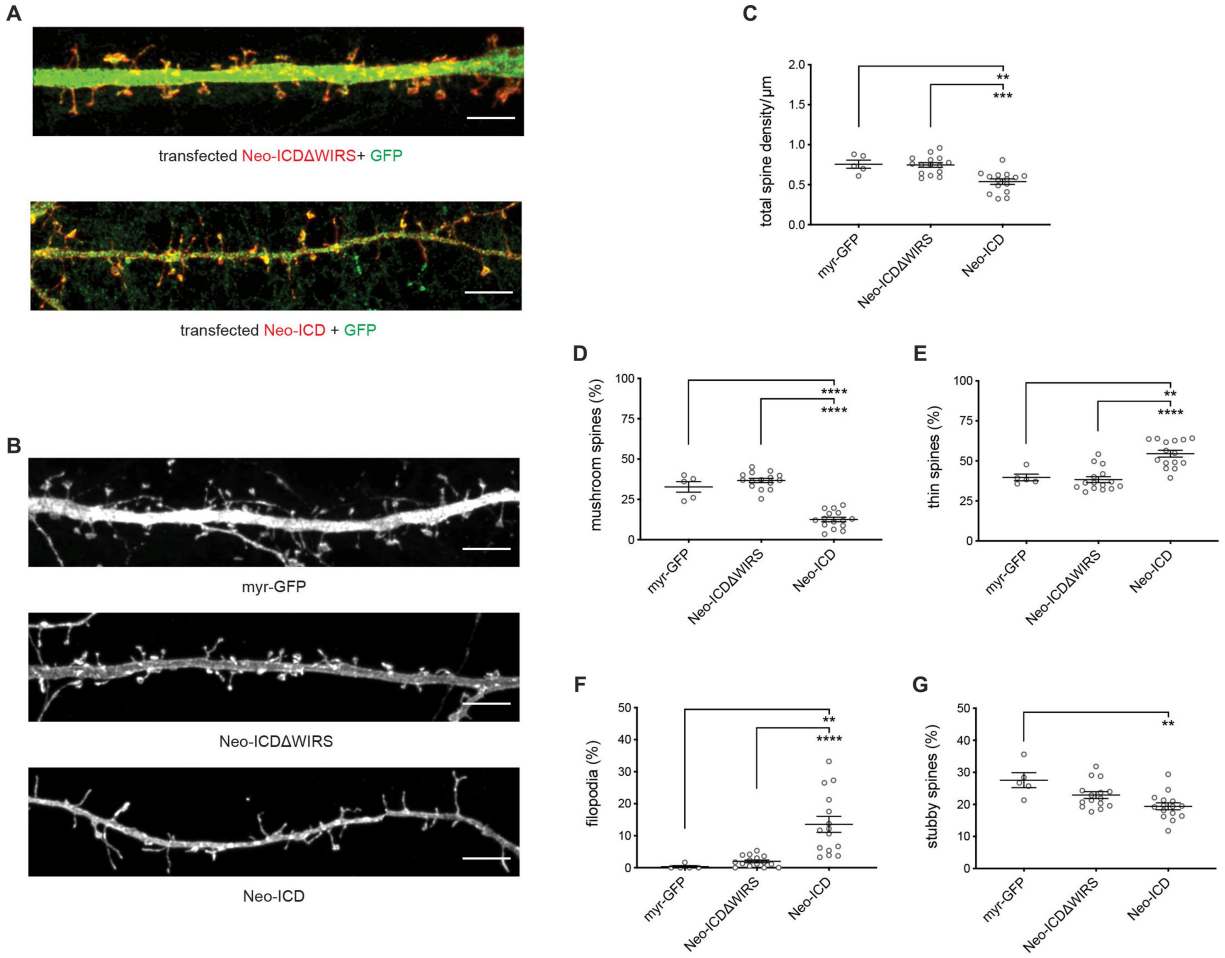
Dendritic spine growth and enlargement depend on the direct interaction between Neogenin and the WRC. **(A)** Neo-ICD and Neo-ICDΔWIRS were concentrated in the heads of mushroom and stubby spines as well as in thin spines and filopodia (GFP; green). **(B)** Representative images of transfected neurons. **(C)** Spine density was decreased by Neo-ICD, but not Neo-ICDΔWIRS [*F*_(2,32)_ = 12.85, *p* < 0.0001]. Neo-ICD decreased the proportion of mushroom spines [*F*_(2,32)_ = 75.93, *p* < 0.0001] **(D)** and increased the proportion of thin spines [*F*_(2,32)_ = 19.39, *p* < 0.0001] **(E)**, filopodia [*F*_(2,32)_ = 14.79, *p* < 0.0001] **(F)** and stubby spines [*F*_(2,32)_ = 7.229, *p* = 0.0026] **(G)**, whereas Neo-ICDΔWIRS had no effect. Neo-ICD, Neo-ICDΔWIRS, *n* = 15 neurons, 3 independent experiments; GFP, *n* = 5 neurons, one experiment; one-way ANOVA, Tukey’s *post hoc* test, mean ± SEM, **p < 0.01, ***p < 0.001, ****p < 0.0001. Scale bars: **(A,B)** 5 μm.

To confirm that Neogenin-WRC interactions are required for spine maturation we used cDNAs encoding the wildtype or WIRS-mutated Neogenin cytoplasmic domain (Neo-ICD, Neo-ICDΔWIRS) containing a myristoylation sequence at the N-terminus to ensure insertion into the plasma membrane. In previous studies we showed that Neo-ICD blocks endogenous Neogenin-WRC interactions and prevents the recruitment of the WRC to adherens junctions whereas Neo-ICDΔWIRS had no effect due to its inability to interact with the Cyfip/Abi binding pocket (O'Leary et al., 2017). After transfection into hippocampal neurons, Neo-ICD and Neo-ICDΔWIRS were predominantly localized to spines (Figure 4A). Spine density analysis revealed that while Neo-ICDΔWIRS had no effect on total spine density, Neo-ICD significantly reduced spine density (Figures 4B,C). This effect was not observed after shRNA depletion probably due to the activity of the residual Neogenin. As expected, we observed no significant differences in the relative proportion of filopodia, thin, mushroom or stubby spines after transfection of Neo-ICDΔWIRS when compared to myristoylated-GFP alone (Figures 4B,D–G). Conversely, Neo-ICD markedly impaired dendritic spine enlargement as indicated by a 66% decrease in mushroom spines and a 42% increase in thin spines relative to neurons expressing Neo-ICDΔWIRS (Figures 4B,D,E). In addition, the expression of Neo-ICD, but not Neo-ICDΔWIRS, significantly increased the proportion of filopodia by 6.8-fold and decreased stubby spines by 1.4-fold (Figures 4B,F,G). Together, these data support the hypothesis that a direct interaction between Neogenin and the WRC is required for spine growth.

**Figure 5:**
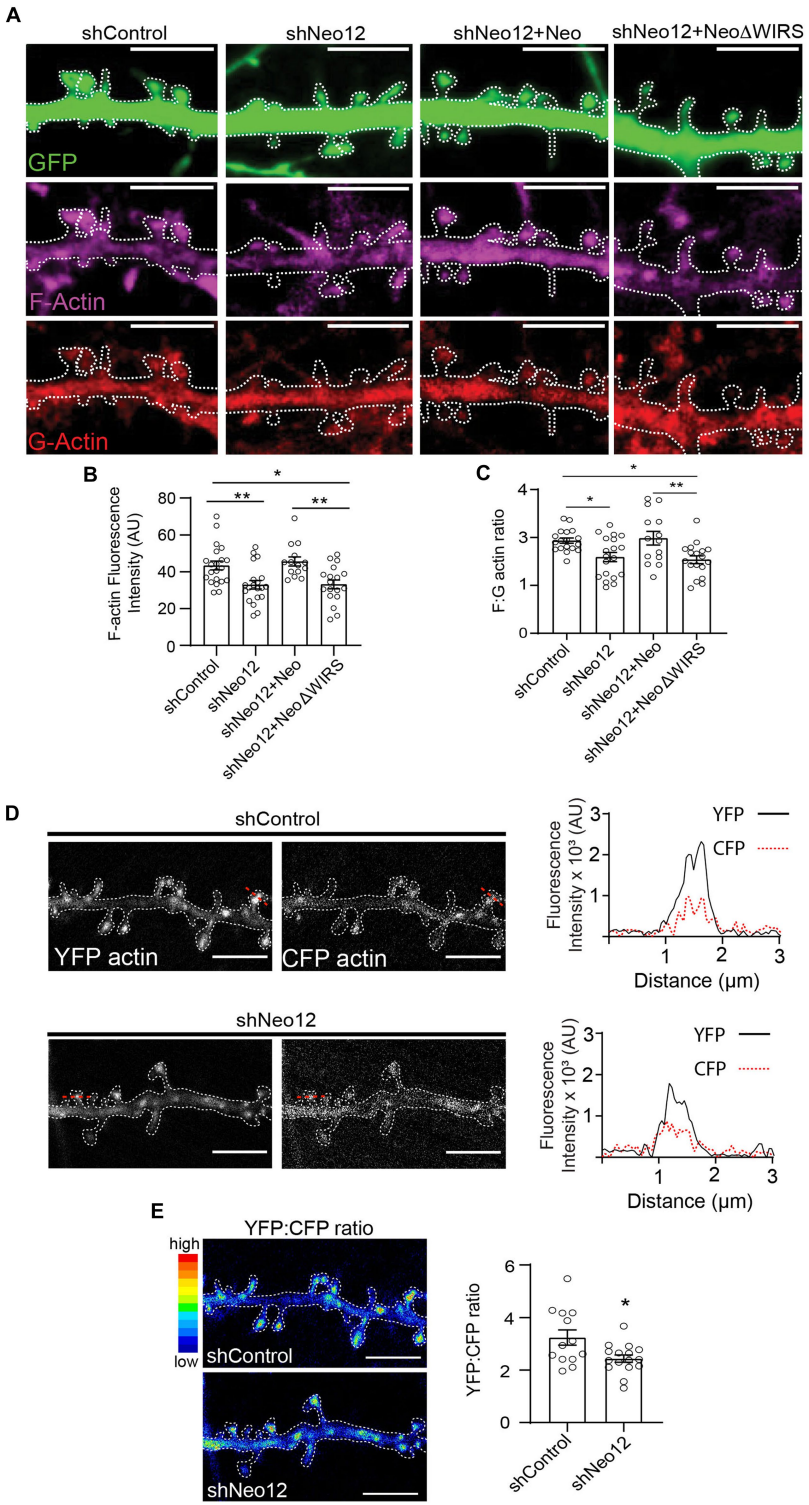
Neogenin-WRC interactions are required for actin polymerization in spines. **(A)** Representative images of spines showing G-actin labeled with DNase 1 (red) and F-actin labeled with phalloidin (magenta; GFP, green). **(B)** Quantification of F-actin (phalloidin) fluorescence intensity in spines (AU, arbitrary units). *n* = 14–19 neurons, 3 independent experiments [*F*_(3,69)_ = 7.50, *p* = 0.0002], one-way ANOVA, Tukey’s *post hoc* test. **(C)** F:G ratios after co-transfection with GFP, shNeo12 and Neo or NeoΔWIRS. *n* = 14–19 neurons, 4 independent experiments [*F*_(3,65)_ = 5.971, *p* = 0.0012], one-way ANOVA, Tukey’s *post hoc* test. **(D)** FRET analysis: representative pseudo-colored images of neurons transfected with the CFP-actin/YFP-actin donor/acceptor pair. Plots show representative profiles of CFP-actin (donor, 485 nm) emission and the FRET signal from YFP-actin (acceptor, 530 nm) generated in a single spine indicated by the red line. **(E)** The YFP:CFP fluorescence intensity ratio was decreased after Neogenin depletion, indicating reduced actin polymerization. *n* = 13–16 neurons, 3 independent experiments [*F*_(12,15)_ = 3.653, *p* = 0.0203], Student’s *t*-test, **(A,B)** mean ± SEM; *p < 0.05,**p < 0.01. Scale bar: **(A,D,E)** 5 μm.

To investigate whether Neogenin is required for branched actin remodeling we determined the level of F-actin in spines after the depletion of Neogenin by quantifying the fluorescence intensity of phalloidin-Alexa 647. We observed a 24% reduction in F-actin in spines after depletion of Neogenin and F-actin was restored to control levels by co-transfection with wildtype Neo but not NeoΔWIRS (Figures 5A,B). Derivation of the F-actin to G-actin (globular actin) ratio from the fluorescence intensities of phalloidin and DNase 1-Alexa-594 which specifically labels G-actin confirmed a 19% reduction in the F:G-actin ratio (Figure 5C). Again, the WIRS mutant was unable to rescue the deficit.

We then directly addressed whether Neogenin promotes actin polymerization by performing fluorescence resonance energy transfer (FRET) imaging in spines from live neurons after co-transfecting shRNAs and cDNAs encoding the CFP-actin/YFP-actin donor/acceptor pair (Okamoto et al., 2004). In this system, a FRET signal is detected when the CFP-actin (donor) and YFP-actin (acceptor) monomers are closely associated, indicating an increase in actin polymerization. Consistent with previous experiments (Okamoto et al., 2004), imaging of hippocampal spines revealed that the FRET signal from YFP-actin was concentrated and readily detectable in spines in the presence of Neogenin and was weaker in dendritic shafts (Figure 5D). After Neogenin depletion the FRET signal was significantly reduced as confirmed by the 20% decrease in the YFP:CFP ratio (Figure 5E). As this correlates well with our F:G-actin ratio analysis above, we conclude that Neogenin-WRC interactions are required to maintain F-actin levels in the spine.

### 3.3. RGMa regulates dendritic spine enlargement via the Neogenin-WRC pathway

**Figure 6:**
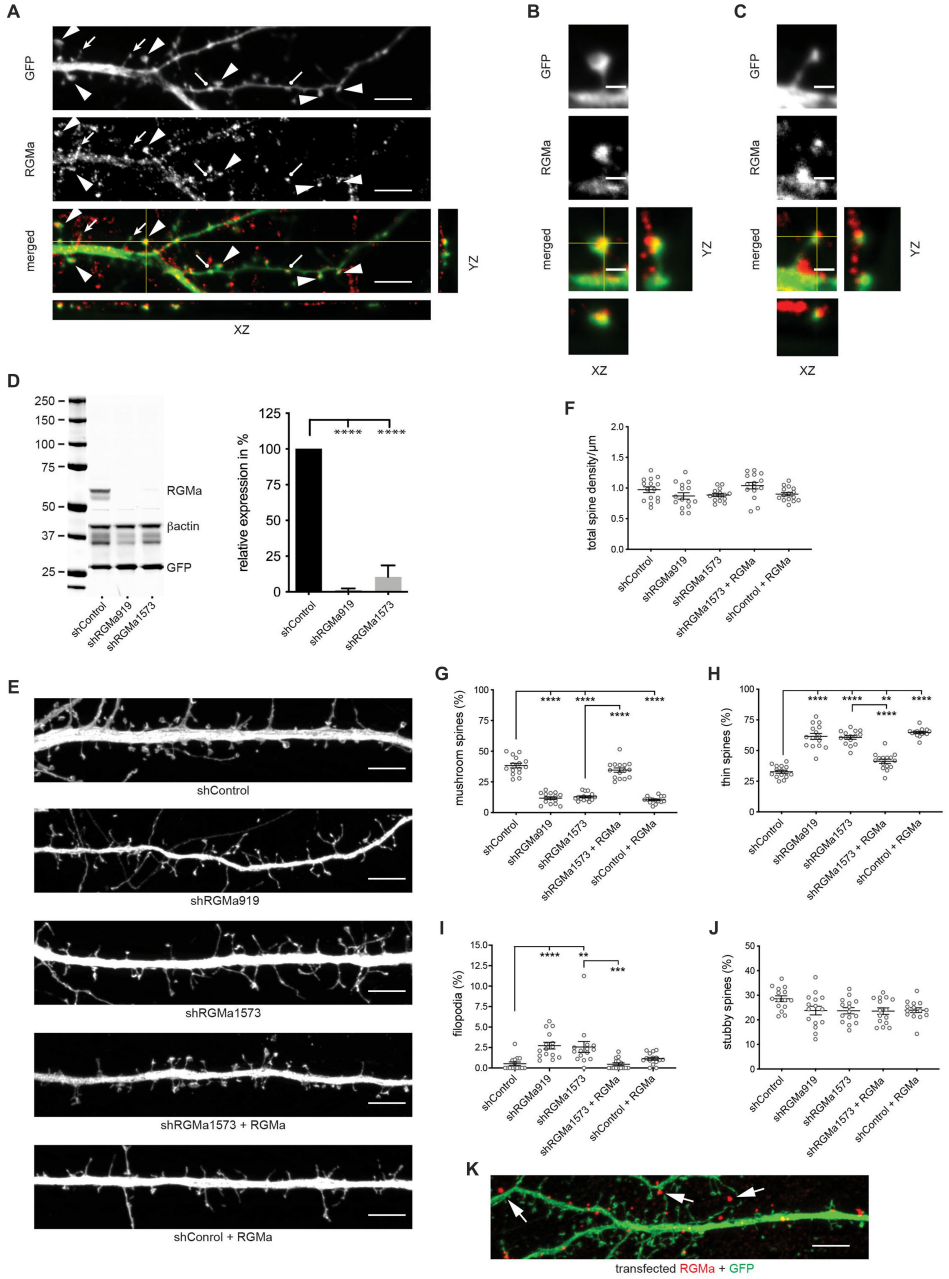
RGMa regulates dendritic spine enlargement. **(A)** Endogenous RGMa was expressed in dendritic spines in DIV14 hippocampal neurons (arrowheads, mushroom spines; filled arrows, thin spines; round-headed arrows, stubby spines) and was detected in the heads of mushroom spines **(B)** and the tips of thin spines **(C)**. **(D)** Immunoblot and quantification: shRNAs efficiently reduced RGMa expression in HEK293T cells, *n* = 3, one-way ANOVA, Tukey’s *post hoc* test; shRGMa919, shRGMa1573, *p* < 0.0001 [*F*_(2,6)_ = 131.3, *p* < 0.0001]. **(E)** Representative images of neurons transfected with GFP, shRGMa1573, and RGMa. Depletion of RGMa did not affect spine density [*F*_(4,70)_ = 2.644, *p* = 0.0406] **(F)**, but decreased the proportion of mushroom spines [*F*_(4,70)_ = 102.5, *p* < 0.0001] **(G)** and increased the proportion of thin spines [*F*_(4,70)_ = 76.20, *p* < 0.0001] **(H)** and filopodia (*p* = 0.0013) **(I)**. This phenotype was rescued by transfected RGMa. The proportion of stubby spines [*F*_(4,70)_ = 2.665, *p* = 0.0394] **(J)** was not affected. *n* = 15 neurons, 3 independent experiments. Mushroom, thin, stubby spines: one-way ANOVA, Tukey’s *post hoc* test. Filopodia: Kruskal–Wallis test, Dunn’s *post hoc* test. **(K)** RGMa was seen in dendrites and spines after transfection of shRNA-resistant myc-tagged RGMa (arrows). **(D,F–J)** mean ± SEM, **p < 0.01, ***p < 0.001, ****p < 0.0001. Scale bars: **(A,E,K)** 5 μm; **(B,C)** 1 μm.

As RGMa is the Neogenin ligand triggering branched actin nucleation via the WRC in epithelial adherens junctions, and like Neogenin, it is expressed throughout the hippocampus, including the dentate gyrus (Lee et al., 2016; Isaksen et al., 2020), we next asked whether RGMa was also required for spine enlargement. Immunolabeling of DIV14 hippocampal neurons revealed that, as for Neogenin, endogenous RGMa was concentrated in the heads of thin and mushroom spines (Figures 6A–C). To determine if RGMa plays a role in spinogenesis RGMa-specific shRNAs (shRGMa919, shRGMa1573) were expressed in hippocampal neurons. Co-transfection of shRNAs and mouse RGMa into HEK293T cells resulted in 90% RGMa depletion (Figure 6D). In neurons no significant change in spine density was observed after RGMa knockdown (Figures 6E,F). In contrast, RGMa depletion replicated the Neogenin phenotype whereby RGMa shRNAs significantly reduced the proportion of mushroom spines by 66–69% (Figures 6E,G) and dramatically enhanced the proportion of thin spines (85–88% increase) (Figures 6E,H). The number of filopodia was also increased by 5-fold, whereas the number of stubby spines remained unchanged (Figures 6E,I,J). The spine maturation phenotype was fully rescued after co-expression of RNAi-resistant RGMa (Figures 6E,G–I,K). Interestingly, the overexpression of RGMa in control cells substantially suppressed mushroom spine formation and increased the proportion of thin spines (Figures 6E,G,H). Therefore, both loss and overexpression of RGMa have a profound effect on spine maturation, indicating that spine enlargement is dependent on tightly regulated levels of RGMa.

**Figure 7:**
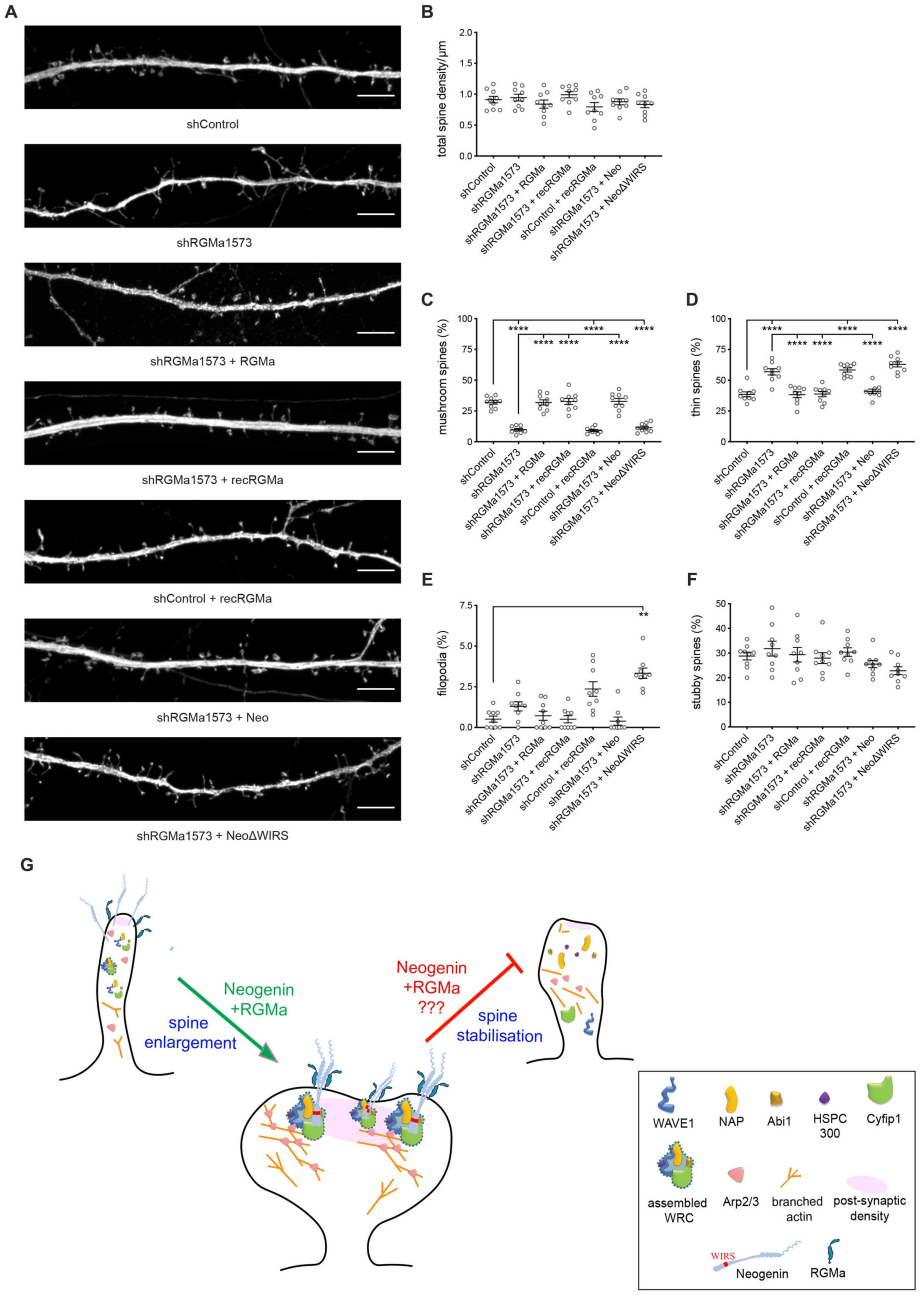
RGMa controls dendritic spine enlargement via the Neogenin-WRC pathway. **(A)** Representative images of neurons transfected with GFP, shRGMa, and RGMa or treated with recombinant RGMa (recRGMa). Depletion of RGMa did not affect spine density [*F*_(6,56)_ = 1.459, *p* = 0.2092] **(B)**, but decreased the proportion of mushroom spines [*F*_(6,56)_ = 46.77, *p* < 0.0001] **(C)** and increased the proportion of thin spines [*F*_(6,56)_ = 27.24, *p* < 0.0001] **(D)**. This phenotype was rescued by transfected RNAi-resistant RGMa or recRGMa. Wildtype Neo but not NeoΔWIRS rescued the phenotype. The proportions of filopodia **(E)** and stubby spines [*F*_(6,56)_ = 1.966, *p* = 0.0861] **(F)** were not affected. Mushroom, thin, stubby spines: one-way ANOVA, Tukey’s *post hoc* test. Filopodia: Kruskal–Wallis test, Dunn’s *post hoc* test. *n* = 9 neurons, 3 independent experiments, mean ± SEM, **p < 0.01, ****p < 0.0001. **(G)** Model: RGMa-Neogenin interactions recruit the WRC to the PSD promoting WRC-dependent actin polymerization and spine enlargement. Whether this pathway is also involved in maintaining spine volume is unknown. Scale bar: **(A)** 5 μm.

RGMa is a glycosylphosphatidylinositol (GPI)-linked protein that can act in cis or trans after cleavage from the membrane through the action of proprotein convertases (Tassew et al., 2012). We therefore investigated whether the membrane-bound or extracellular form of RGMa was required for spine development. To do so, we repeated the RGMa RNAi experiment and determined whether recombinant RGMa could rescue the spinogenesis defect. We found that recombinant and co-transfected RGMa were equally efficient in restoring spine development after RGMa depletion as indicated by the concomitant increase in mushroom spines and reduction in thin spines (Figures 7A,C,D). Spine density and the number of stubby spines and filopodia remained unchanged (Figures 7A,B,E,F). In addition, as seen for transfected RGMa, recombinant RGMa in the absence of RGMa shRNA inhibited the maturation of thin spines (Figures 7A,C,D), thereby confirming that tight control of extracellular RGMa levels is required for successful spine enlargement.

Finally, to demonstrate that RGMa is the cognate ligand for Neogenin driving spine enlargement, we also co-transfected shRGMa and wildtype Neo or NeoΔWIRS. The proportions of thin and mushroom spines were restored to normal levels by wildtype Neo whereas the WIRS mutant was unable to rescue the spine phenotype (Figures 7A,C,D), confirming that during spinogenesis RGMa lies upstream of Neogenin and the WRC. These data provide strong evidence that RGMa acts in trans to activate the Neogenin-WRC pathway and drive spine growth and enlargement.

## 4. Discussion

Structural plasticity, the ability of dendritic spines to change their volume in response to synaptic stimulation in the hippocampus, is an essential determinant of synaptic strength and LTP induction (Matsuzaki et al., 2004; Harvey and Svoboda, 2007; Bourne and Harris, 2011; Watson et al., 2016). In the motor cortex, spine formation allows the functional rewiring of circuits during motor learning (Xu et al., 2009; Albarran et al., 2021). Branched actin polymerization is a major force promoting spine head enlargement and therefore sustains structural plasticity (Hotulainen et al., 2009; Korobova and Svitkina, 2010; Bosch et al., 2014; Chazeau et al., 2014). The WRC is now recognized as a pivotal branched actin regulator controlling spine morphology. Although tight spatiotemporal control of the WRC is critical for spine expansion (Grove et al., 2004; Kim et al., 2006; Soderling et al., 2007; De Rubeis et al., 2013; Hazai et al., 2013; Pathania et al., 2014; Davenport et al., 2019), we currently have only a rudimentary understanding of the molecular mechanisms that govern the recruitment of the WRC to the post-synaptic density and its subsequent activation. Here we identify a novel role for the RGMa receptor, Neogenin, as a principal component of the branched actin nucleation machinery driving spine enlargement in hippocampal neurons. We demonstrate that Neogenin recruits the WRC to the spine head to facilitate Arp2/3-dependent branched actin polymerization and spine enlargement. We further reveal that RGMa is the cognate ligand for Neogenin in the spine where it is required to promote WRC activation, demonstrating for the first time a role for RGMa in spine morphogenesis. This study also provides mechanistic insight into Neogenin’s emerging role in LTP induction.

During spine morphogenesis thin spines transform into more long-lived mushroom spines enabling the formation of strong synaptic connections with the opposing axonal bouton (Ziv and Smith, 1996; Lohmann and Bonhoeffer, 2008; Yuste, 2013). This transition is dependent on the rapid assembly of the branched actin cytoskeleton and is correlated with an increase in the size of the post-synaptic density (Hotulainen et al., 2009; Korobova and Svitkina, 2010; Bosch et al., 2014; Chazeau et al., 2014). We demonstrate here that Neogenin is a critical actin polymerization regulator due to its ability to occupy the highly conserved binding pocket within the WRC comprising the Cyfip1 and Abi subunits. We found that the depletion of Neogenin dramatically increased the proportion of thin spines and filopodia while decreasing the number of mushroom spines, and that wildtype Neogenin but not the WIRS mutant was able to rescue the immature spine phenotype. Moreover, the loss of Neogenin inhibited actin polymerization, an effect that was not restored by the WIRS mutant. These findings are in line with the observation that WAVE1 or Cyfip1 depletion results in the loss of mature spines (Kim et al., 2006; Soderling et al., 2007; De Rubeis et al., 2013; Pathania et al., 2014; Davenport et al., 2019). We therefore conclude that the direct interaction between Neogenin and the WRC promotes branched actin nucleation, facilitating spine enlargement. These findings offer an explanation for the impairment of spine maturation previously reported after genetic deletion of Neogenin (Sun D. et al., 2018; Sun X.-D. et al., 2018). As Neogenin continues to be expressed in mature mushroom spines well after the developmental phase, it is also possible that Neogenin-WRC interactions maintain the mature spine morphology through persistent actin remodeling (Figure 7G).

Upon synaptic stimulation, the WRC localizes to the post-synaptic density in close proximity to its upstream activator Rac1 where it activates Arp2/3-mediated branched actin nucleation (Kim et al., 2006; Soderling et al., 2007; De Rubeis et al., 2013; Chazeau et al., 2014; Chen et al., 2017). Direct evidence that Neogenin spatially restricts the WRC within the spine membrane in close association with Arp2/3 was provided by the expression of the membrane-bound, cytoplasmic domain, Neo-ICD, which is able to bind and sequester the WRC away from its site of action (O'Leary et al., 2017). We observed that Neo-ICD prevented spine enlargement as seen after Neogenin shRNA depletion, supporting the proposal that Neogenin positions the WRC close to Arp2/3. In contrast, Neo-ICDΔWIRS which is unable to bind the WRC had no effect on spine enlargement. Together, these data lead to a model in which Neogenin is a crucial component of the branched actin nucleation machinery in the expanding spine where it restricts the WRC to the post-synaptic density in close proximity to Arp2/3 (Figure 7G). This conclusion is strongly supported by our previous studies in which Neogenin was found to be essential for the recruitment of the WRC and Arp2/3 to the restricted sites of cadherin adhesion in epithelial cells and cortical neural progenitors (Lee et al., 2016; O'Leary et al., 2017).

Cyfip1 also inhibits mRNA translation in the spine by forming a complex with the Fragile X protein, FMRP, and the translation initiation factor EIF4E (Napoli et al., 2008; De Rubeis et al., 2013). Translational repression is relieved by synaptic activity which triggers Cyfip1 dissociation from the FMRP-EIF4E complex. Cyfip1 then translocates to the post-synaptic density where it is incorporated into the WRC. This dynamic Cyfip1 redistribution between FMRP and the WRC ensures that actin remodeling and protein synthesis are tightly coupled - a fundamental requirement for synaptic plasticity. In pyramidal neurons of the basolateral amygdala Neogenin promotes both the induction and maintenance of LTP (Sun X.-D. et al., 2018). It is also essential for the induction of LTP at the entorhinal to granule cell synapse of the perforant path (Liakath-Ali et al., 2022). However, to date, the downstream Neogenin effectors enabling LTP have not been identified. The ability of Neogenin to anchor the WRC to the membrane adjacent to the post-synaptic density implies that it is an important regulator of Cyfip1 distribution during synaptic enlargement. Our data support the concept that Neogenin constrains Cyfip1 within the WRC, and in doing so, acts to relieve translational repression while concomitantly promoting WRC/Arp2/3-mediated actin remodeling. As such, this model provides a mechanistic basis for understanding Neogenin’s involvement in LTP.

Given our identification of RGMa as the Neogenin ligand required for WRC activation in epithelial cells (Lee et al., 2016), we predicted that RGMa was the cognate ligand required for spine enlargement. Indeed, we demonstrate that as for Neogenin depletion, knockdown of RGMa inhibits spine enlargement, a phenotype that is fully rescued by both recombinant RGMa and wildtype Neogenin. Failure of the Neogenin WIRS mutant to restore spine enlargement confirmed that RGMa lies upstream of the Neogenin-WRC pathway (Figure 7G). We further observed that high concentrations of RGMa also substantially impaired spine head expansion, indicating that WRC activation is sensitive to both high and low RGMa concentrations. The RGMa-Neogenin signaling complex comprises two RGMa and two Neogenin molecules (Bell et al., 2013; Siebold et al., 2017; Robinson et al., 2021). As such, the absence of RGMa or high RGMa concentrations would be expected to prevent the formation of the 2:2 complex. Moreover, RGMa also controls the availability of membrane-bound Neogenin which is susceptible to cleavage by the metalloprotease ADAM17 where a direct interaction between Neogenin and the leucine-rich repeat protein, Lrig2, protects against cleavage (van Erp et al., 2015). However, as RGMa binding to Neogenin disrupts this interaction, high RGMa concentrations are likely to expose Neogenin to ADAM17, leading to its depletion on the post-synaptic membrane. It has been proposed that induction of LTP at the perforant synapse is reliant on the ability of post-synaptic Neogenin to form a trans-synaptic adhesion complex with pre-synaptic Neurexin-1 via its ligand Cerebellin-4 (Liakath-Ali et al., 2022). Our study therefore suggests that Neogenin signal transduction in the spine is tightly regulated by the coordinated activity of these two distinct ligands.

Evidence is now accumulating that the functional clustering of autism spectrum disorder (ASD) genes into a few convergent signaling pathways underpins the etiology of ASD (Parikshak et al., 2013; Iossifov et al., 2014). The frequent occurrence of disruptive mutations in genes regulating spine morphogenesis and synaptic plasticity, particularly those associated with the actin cytoskeleton, strongly implicates actin remodeling pathways in ASD (Parikshak et al., 2013; Iossifov et al., 2014; Sanders et al., 2015). Indeed, several WRC subunit genes, including *NCKAP1* and the WAVE1 gene, *WASF1,* are strongly implicated in ASD (Iossifov et al., 2012, 2014; Ito et al., 2018). Mutations in the *CYFIP1* gene are associated with the severe ASD-related Angelman and Prader-Willi syndromes, as well as ASD and schizophrenia (Bagni and Zukin, 2019). Neogenin has also been implicated in ASD where a mutation that deletes the WIRS motif has been identified in an ASD patient (Siu et al., 2016). We therefore propose that the Neogenin-WRC pathway constitutes a cluster of ASD genes whose convergent activity plays a central role in actin remodeling, spine morphogenesis and synaptic plasticity.

## Data availability statement

The raw data supporting the conclusions of this article will be made available by the authors, without undue reservation.

## Ethics statement

Ethical approval was not required for the studies on humans in accordance with the local legislation and institutional requirements because only commercially available established cell lines were used. The animal study was approved by the Anatomical Biosciences Animal Ethics Committee of the University of Queensland. The study was conducted in accordance with the local legislation and institutional requirements.

## Author contributions

KS: Conceptualization, Formal analysis, Investigation, Methodology, Writing – review & editing. BS: Conceptualization, Formal analysis, Investigation, Methodology, Writing – review & editing. VL: Methodology, Supervision, Writing – review & editing. EO’B: Methodology, Project administration, Writing – review & editing. CF: Conceptualization, Funding acquisition, Methodology, Supervision, Writing – review & editing. HC: Conceptualization, Formal analysis, Funding acquisition, Methodology, Supervision, Writing – original draft, Writing – review & editing.

## References

[ref1] Albarran E. Raissi A. Jaidar O. Shatz C. J. Ding J. B. (2021). Enhancing motor learning by increasing the stability of newly formed dendritic spines in the motor cortex. Neuron 109, 3298–3311. doi: 10.1016/j.neuron.2021.07.030, PMID: 34437845 PMC8542616

[ref2] Bagni C. Zukin R. S. (2019). A synaptic perspective of fragile X syndrome and autism spectrum disorders. Neuron 101, 1–19. doi: 10.1016/j.neuron.2019.02.041 30897358 PMC9628679

[ref3] Bell C. H. Healey E. van Erp S. Bishop B. Tang C. Gilbert R. J. C.. (2013). Structure of the repulsive guidance molecule (RGM)-Neogenin signaling hub. Science 341, 77–80. doi: 10.1126/science.1232322, PMID: 23744777 PMC4730555

[ref4] Bosch M. Castro J. Saneyoshi T. Matsuno H. Sur M. Hayashi Y. (2014). Structural and molecular remodeling of dendritic spine substructures during long-term potentiation. Neuron 82, 444–459. doi: 10.1016/j.neuron.2014.03.021, PMID: 24742465 PMC4281348

[ref5] Bourne J. N. Harris K. M. (2011). Coordination of size and number of excitatory and inhibitory synapses results in a balanced structural plasticity along mature hippocampal CA1 dendrites during LTP. Hippocampus 21, 354–373. doi: 10.1002/hipo.20768, PMID: 20101601 PMC2891364

[ref6] Chazeau A. Mehidi A. Nair D. Gautier J. J. Leduc C. Chamma I.. (2014). Nanoscale segregation of actin nucleation and elongation factors determines dendritic spine protrusion. EMBO J. 33, 2745–2764. doi: 10.15252/embj.201488837, PMID: 25293574 PMC4282554

[ref7] Chen B. Brinkmann K. Chen Z. Pak C. W. Liao Y. Shi S.. (2014). The WAVE regulatory complex links diverse receptors to the actin cytoskeleton. Cell 156, 195–207. doi: 10.1016/j.cell.2013.11.048, PMID: 24439376 PMC4059610

[ref8] Chen B. Chou H.-T. Brautigam C. A. Xing W. Yang S. Henry L.. (2017). Rac1 GTPase activates the WAVE regulatory complex through two distinct binding sites. eLife 6:W529. doi: 10.7554/elife.29795 PMC5614565 28949297

[ref9] Davenport E. C. Szulc B. R. Drew J. Taylor J. Morgan T. Higgs N. F.. (2019). Autism and schizophrenia-associated CYFIP1 regulates the balance of synaptic excitation and inhibition. Cell Rep. 26, 2037–2051. doi: 10.1016/j.celrep.2019.01.092, PMID: 30784587 PMC6381785

[ref10] De Rubeis S. Pasciuto E. Li K. W. Fernandez E. Di Marino D. Buzzi A.. (2013). CYFIP1 coordinates mRNA translation and cytoskeleton remodeling to ensure proper dendritic spine formation. Neuron 79, 1169–1182. doi: 10.1016/j.neuron.2013.06.039, PMID: 24050404 PMC3781321

[ref11] De Vries M. Cooper H. M. (2008). Emerging roles for Neogenin and its ligands in CNS development. J. Neurochem. 106, 1483–1492. doi: 10.1111/j.1471-4159.2008.05485.x, PMID: 18485097

[ref12] Dull T. Zufferey R. Kelly M. Mandel R. J. Nguyen M. Trono D.. (1998). A third-generation lentivirus vector with a conditional packaging system. J. Virol. 72, 8463–8471. doi: 10.1128/JVI.72.11.8463-8471.1998, PMID: 9765382 PMC110254

[ref13] Gad J. M. Keeling S. L. Wilks A. F. Tan S. S. Cooper H. M. (1997). The expression patterns of guidance receptors, DCC and Neogenin, are spatially and temporally distinct throughout mouse embryogenesis. Dev. Biol. 192, 258–273. doi: 10.1006/dbio.1997.8756, PMID: 9441666

[ref14] Grove M. Demyanenko G. Echarri A. Zipfel P. A. Quiroz M. E. Rodriguiz R. M.. (2004). ABI2-deficient mice exhibit defective cell migration, aberrant dendritic spine morphogenesis, and deficits in learning and memory. Mol. Cell. Biol. 24, 10905–10922. doi: 10.1128/MCB.24.24.10905-10922.2004, PMID: 15572692 PMC533973

[ref15] Harvey C. D. Svoboda K. (2007). Locally dynamic synaptic learning rules in pyramidal neuron dendrites. Nature 450, 1195–1200. doi: 10.1038/nature06416, PMID: 18097401 PMC3425382

[ref16] Hazai D. Szudoczki R. Ding J. Soderling S. H. Weinberg R. J. Sotonyi P.. (2013). Ultrastructural abnormalities in CA1 hippocampus caused by deletion of the actin regulator WAVE-1. PLoS One 8:e75248. doi: 10.1371/journal.pone.0075248, PMID: 24086480 PMC3783472

[ref17] Honkura N. Matsuzaki M. Noguchi J. Ellis-Davies G. C. Kasai H. (2008). The subspine organization of actin fibers regulates the structure and plasticity of dendritic spines. Neuron 57, 719–729. doi: 10.1016/j.neuron.2008.01.013, PMID: 18341992

[ref18] Hotulainen P. Llano O. Smirnov S. Tanhuanpaa K. Faix J. Rivera C.. (2009). Defining mechanisms of actin polymerization and depolymerization during dendritic spine morphogenesis. J. Cell Biol. 185, 323–339. doi: 10.1083/jcb.200809046, PMID: 19380880 PMC2700375

[ref19] Huang Z. Sun D. Hu J. X. Tang F. L. Lee D. H. Wang Y.. (2016). Neogenin promotes BMP2 activation of YAP and Smad1 and enhances astrocytic differentiation in developing mouse neocortex. J. Neurosci. 36, 5833–5849. doi: 10.1523/JNEUROSCI.4487-15.2016, PMID: 27225772 PMC4879200

[ref20] Huang Z. Xiong W. C. (2016). Neogenin-YAP signaling in neocortical astrocytic differentiation. Neurogenesis 3:e1248735. doi: 10.1080/23262133.2016.1248735, PMID: 28405584 PMC5384612

[ref21] Iossifov I. O’Roak B. J. Sanders S. J. Ronemus M. Krumm N. Levy D.. (2014). The contribution of de novo coding mutations to autism spectrum disorder. Nature 515, 1–17. doi: 10.1038/nature13908 PMC4313871 25363768

[ref22] Iossifov I. Ronemus M. Levy D. Wang Z. Hakker I. Rosenbaum J.. (2012). De novo gene disruptions in children on the autistic spectrum. Neuron 74, 285–299. doi: 10.1016/j.neuron.2012.04.009, PMID: 22542183 PMC3619976

[ref23] Isaksen T. J. Fujita Y. Yamashita T. (2020). Repulsive guidance molecule A suppresses adult neurogenesis. Stem Cell Reports 14, 677–691. doi: 10.1016/j.stemcr.2020.03.003, PMID: 32243839 PMC7160374

[ref24] Ito Y. Carss K. J. Duarte S. T. Hartley T. Keren B. Kurian M. A.. (2018). De novo truncating mutations in WASF1 cause intellectual disability with seizures. Am. J. Hum. Genet. 103, 144–153. doi: 10.1016/j.ajhg.2018.06.001, PMID: 29961568 PMC6037130

[ref25] Kam J. W. K. Dumontier E. Baim C. Brignall A. C. Silva D. M. D. Cowan M.. (2016). RGMB and Neogenin control cell differentiation in the developing olfactory epithelium. Development 143, 1534–1546. doi: 10.1242/dev.118638, PMID: 27143755

[ref26] Keeling S. L. Gad J. M. Cooper H. M. (1997). Mouse Neogenin, a DCC-like molecule, has four splice variants and is expressed widely in the adult mouse and during embryogenesis. Oncogene 15, 691–700. doi: 10.1038/sj.onc.1201225, PMID: 9264410

[ref27] Kim Y. Sung J. Y. Ceglia I. Lee K.-W. Ahn J.-H. Halford J. M.. (2006). Phosphorylation of WAVE1 regulates actin polymerization and dendritic spine morphology. Nature 442, 814–817. doi: 10.1038/nature04976, PMID: 16862120

[ref28] Korobova F. Svitkina T. (2010). Molecular architecture of synaptic actin cytoskeleton in hippocampal neurons reveals a mechanism of dendritic spine morphogenesis. Mol. Biol. Cell 21, 165–176. doi: 10.1091/mbc.e09-07-0596 19889835 PMC2801710

[ref29] Lanoue V. Langford M. White A. Sempert K. Fogg L. Cooper H. M. (2017). The Wnt receptor Ryk is a negative regulator of mammalian dendrite morphogenesis. Sci. Rep. 7:5965. doi: 10.1016/j.ydbio.2018.12.005 28729735 PMC5519545

[ref30] Lee N. Fok K. White A. Wilson N. O'Leary C. Cox H.. (2016). Neogenin recruitment of the WAVE regulatory complex maintains adherens junction stability and tension. Nat. Comms. 7:11082. doi: 10.1038/ncomms11082 PMC4821876 27029596

[ref31] Liakath-Ali K. Polepalli J. S. Lee S. J. Cloutier J. F. Sudhof T. C. (2022). Transsynaptic cerebellin 4-neogenin 1 signaling mediates LTP in the mouse dentate gyrus. Proc. Natl. Acad. Sci. U. S. A. 119:e2123421119. doi: 10.1073/pnas.2123421119, PMID: 35544694 PMC9171784

[ref32] Lohmann C. Bonhoeffer T. (2008). A role for local calcium signaling in rapid synaptic partner selection by dendritic filopodia. Neuron 59, 253–260. doi: 10.1016/j.neuron.2008.05.025, PMID: 18667153

[ref33] Lois C. Hong E. J. Pease S. Brown E. J. Baltimore D. (2002). Germline transmission and tissue-specific expression of transgene delivery by Lenti-virus vectors. Science 295, 868–872. doi: 10.1126/science.1067081, PMID: 11786607

[ref34] Matsuzaki M. Honkura N. Ellis-Davies G. C. Kasai H. (2004). Structural basis of long-term potentiation in single dendritic spines. Nature 429, 761–766. doi: 10.1038/nature02617, PMID: 15190253 PMC4158816

[ref35] Napoli I. Mercaldo V. Boyl P. P. Eleuteri B. Zalfa F. De Rubeis S.. (2008). The fragile X syndrome protein represses activity-dependent translation through CYFIP1, a new 4E-BP. Cell 134, 1042–1054. doi: 10.1016/j.cell.2008.07.031, PMID: 18805096

[ref36] Okamoto K. Nagai T. Miyawaki A. Hayashi Y. (2004). Rapid and persistent modulation of actin dynamics regulates postsynaptic reorganization underlying bidirectional plasticity. Nat. Neurosci. 7, 1104–1112. doi: 10.1038/nn1311, PMID: 15361876

[ref37] O'Leary C. J. Bradford D. Chen M. White A. Blackmore D. G. Cooper H. M. (2015). The netrin/RGM receptor, Neogenin, controls adult neurogenesis by promoting neuroblast migration and cell cycle exit. Stem Cells 33, 503–514. doi: 10.1002/stem.1861, PMID: 25308084

[ref38] O'Leary C. Cole S. J. Langford M. Hewage J. White A. Cooper H. M. (2013). RGMa regulates cortical interneuron migration and differentiation. PLoS One 8:e81711. doi: 10.1371/journal.pone.0081711, PMID: 24312340 PMC3842424

[ref39] O'Leary C. J. Nourse C. C. Lee N. K. White A. Langford M. Sempert K.. (2017). Neogenin recruitment of the WAVE regulatory complex to ependymal and radial progenitor adherens junctions prevents hydrocephalus. Cell Rep. 20, 370–383. doi: 10.1016/j.celrep.2017.06.051, PMID: 28700939

[ref40] Parikshak N. N. Luo R. Zhang A. Won H. Lowe J. K. Chandran V.. (2013). Integrative functional genomic analyses implicate specific molecular pathways and circuits in autism. Cell 155, 1008–1021. doi: 10.1016/j.cell.2013.10.031, PMID: 24267887 PMC3934107

[ref41] Pathania M. Davenport E. C. Muir J. Sheehan D. F. López-Doménech G. Kittler J. T. (2014). The autism and schizophrenia associated gene CYFIP1 is critical for the maintenance of dendritic complexity and the stabilization of mature spines. Transl. Psychiatry 4:e374. doi: 10.1038/tp.2014.16, PMID: 24667445 PMC3966042

[ref42] Rajagopalan S. Deitinghoff L. Davis D. Conrad S. Skutella T. Chedotal A.. (2004). Neogenin mediates the action of repulsive guidance molecule. Nat. Cell Biol. 6, 756–762. doi: 10.1038/ncb1156, PMID: 15258590

[ref43] Robinson R. A. Griffiths S. C. van de Haar L. L. Malinauskas T. van Battum E. Y. Zelina P.. (2021). Simultaneous binding of guidance cues NET1 and RGM blocks extracellular NEO1 signaling. Cell 184, 2103–2120. doi: 10.1016/j.cell.2021.02.045, PMID: 33740419 PMC8063088

[ref44] Sanders S. J. He X. Willsey A. J. Ercan-Sencicek A. G. Samocha K. E. Cicek A. E.. (2015). Insights into autism spectrum disorder genomic architecture and biology from 71 risk loci. Neuron 87, 1215–1233. doi: 10.1016/j.neuron.2015.09.016, PMID: 26402605 PMC4624267

[ref45] Siebold C. Yamashita T. Monnier P. P. Mueller B. K. Pasterkamp R. J. (2017). RGMs: structural insights, molecular regulation, and downstream signaling. Trends Cell Biol. 27, 365–378. doi: 10.1016/j.tcb.2016.11.009, PMID: 28007423 PMC5404723

[ref46] Siu W.-K. Lam C.-W. Gao W.-W. Vincent Tang H.-M. Jin D.-Y. Mak C. M. (2016). Unmasking a novel disease gene NEO1 associated with autism spectrum disorders by a hemizygous deletion on chromosome 15 and a functional polymorphism. Behav. Brain Res. 300, 135–142. doi: 10.1016/j.bbr.2015.10.041 26518331

[ref47] Soderling S. H. Guire E. S. Kaech S. White J. Zhang F. Schutz K.. (2007). A WAVE-1 and WRP signaling complex regulates spine density, synaptic plasticity, and memory. J. Neurosci. 27, 355–365. doi: 10.1523/JNEUROSCI.3209-06.2006, PMID: 17215396 PMC3740594

[ref48] Spence E. F. Kanak D. J. Carlson B. R. Soderling S. H. (2016). The Arp2/3 complex is essential for distinct stages of spine synapse maturation, including synapse unsilencing. J. Neurosci. 36, 9696–9709. doi: 10.1523/JNEUROSCI.0876-16.2016, PMID: 27629719 PMC5039249

[ref49] Sun X.-D. Chen W.-B. Sun D. Huang J. Li Y.-Q. Pan J.-X.. (2018). Neogenin in amygdala for neuronal activity and information processing. J. Neurosci. 38, 9600–9613. doi: 10.1523/JNEUROSCI.0433-18.2018 30228230 PMC6209834

[ref50] Sun D. Sun X.-D. Zhao L. Lee D.-H. Hu J.-X. Tang F.-L.. (2018). Neogenin, a regulator of adult hippocampal neurogenesis, prevents depressive-like behavior. Cell Death Dis. 9:8. doi: 10.1038/s41419-017-0019-2 29311593 PMC5849041

[ref51] Tassew N. G. Charish J. Seidah N. G. Monnier P. P. (2012). SKI-1 and Furin generate multiple RGMa fragments that regulate axonal growth. Dev. Cell 22, 391–402. doi: 10.1016/j.devcel.2011.11.022, PMID: 22340500

[ref52] van Erp S. van den Heuvel D. M. A. Fujita Y. Robinson R. A. Hellemons A. J. C. G. M. Adolfs Y.. (2015). Lrig2 negatively regulates ectodomain shedding of axon guidance receptors by ADAM proteases. Dev. Cell 35, 537–552. doi: 10.1016/j.devcel.2015.11.008, PMID: 26651291

[ref53] Watson D. J. Ostroff L. Cao G. Parker P. H. Smith H. Harris K. M. (2016). LTP enhances synaptogenesis in the developing hippocampus. Hippocampus 26, 560–576. doi: 10.1002/hipo.22536, PMID: 26418237 PMC4811749

[ref54] Wilson N. H. Key B. (2006). Neogenin interacts with RGMa and netrin-1 to guide axons within the embryonic vertebrate forebrain. Dev. Biol. 296, 485–498. doi: 10.1016/j.ydbio.2006.06.018, PMID: 16836993

[ref55] Xu T. Yu X. Perlik A. J. Tobin W. F. Zweig J. A. Tennant K.. (2009). Rapid formation and selective stabilization of synapses for enduring motor memories. Nature 462, 915–919. doi: 10.1038/nature08389, PMID: 19946267 PMC2844762

[ref56] Yuste R. (2013). Electrical compartmentalization in dendritic spines. Annu. Rev. Neurosci. 36, 429–449. doi: 10.1146/annurev-neuro-062111-150455, PMID: 23724997

[ref57] Ziv N. E. Smith S. J. (1996). Evidence for a role of dendritic filopodia in synaptogenesis and spine formation. Neuron 17, 91–102. doi: 10.1016/S0896-6273(00)80283-4, PMID: 8755481

